# Developing refractive management recommendations for patients undergoing cataract surgery: A Delphi study

**DOI:** 10.1111/opo.13069

**Published:** 2022-11-16

**Authors:** Emily Charlesworth, Paul Ursell, Kam Chun Ho, Lisa Keay, David B. Elliott

**Affiliations:** ^1^ Bradford School of Optometry and Vision Science University of Bradford Bradford UK; ^2^ Department of Ophthalmology Epson & St. Helier University NHS Trust Sutton UK; ^3^ Discipline of Optometry and Vision Science University of Canberra Canberra Australian Capital Territory Australia; ^4^ School of Optometry and Vision Science University of New South Wales Sydney New South Wales Australia

**Keywords:** cataract surgery, Delphi study, guidelines, refractive management

## Abstract

**Purpose:**

Currently, there are no UK optometric guidelines regarding the pre and postoperative refractive management of patients undergoing cataract surgery. This study used a Delphi method to gain consensus on best practice.

**Methods:**

Eighteen recommendations targeted areas of concern/variability in advice that were highlighted in an earlier focus group study of refractive management for patients who had received cataract surgery. These covered three topics: preoperative target refraction discussions, postoperative refractive management and driving advice postoperatively. The recommendations were then developed using evidence from optometry and ophthalmology clinical expertise and the research literature. Eighteen recommendations underwent a process of agreement and modification using a Delphi study consisting of a panel of 22 highly experienced optometrists (*N* = 11, 25 years mean clinical experience) and ophthalmologists (*N* = 11, 17 years mean clinical experience) who rated and commented upon the importance and feasibility of each recommendation. The responses were considered by the research team and the recommendations modified and/or removed prior to a second Delphi round of responses to a modified series of recommendations. Consensus of opinion was defined as greater than 80% of panellists ‘agreed’ or ‘strongly agreed’ on the recommendation for both importance and feasibility.

**Results:**

Fourteen of the 18 recommendations reached consensus in the first round. A second round of the Delphi method saw 17 modified recommendations scored and commented upon by 20 clinicians. Fifteen recommendations reached consensus after two rounds of the Delphi method.

**Conclusions:**

Recommendations to guide the pre and postoperative refractive management of patients undergoing cataract surgery were agreed between highly experienced optometrists and ophthalmologists using a 2‐round Delphi method. Patients would benefit from consistent target refraction discussions, postoperative refractive management and driving advice, and recommendations were of particular benefit to less experienced optometrists.

**Key points**

- Recommendations to guide the pre and postoperative refractive management of patients undergoing cataract surgery were agreed between highly experienced optometrists and ophthalmologists using a 2‐round Delphi method.
- Recommendations included that myopic patients used to reading without glasses should particularly be made aware of the option of a myopic target refraction in monofocal intraocular lens referrals.
- Patients with surgery‐induced anisometropia and/or loss of stereopsis should be advised that this may cause problems with driving and could require a longer period of adaptation.

## INTRODUCTION

Within the UK, approximately 400,000 cataract surgeries are performed per year, with this figure anticipated to increase by 50% in 2035.^1^ Investigating patient experiences of cataract surgery has highlighted several refractive management challenges faced by both patients and practitioners in the preoperative and postoperative period,^2^, ^3^ but there are no UK optometric guidelines regarding best practice refractive management for cataract surgery patients.

Both internationally and nationally, there is agreement that health services, including the provision of cataract surgery, should be people‐centred.^4^, ^5^ Therefore, any attempt to develop guidelines for the refractive management of cataract surgery must include areas of concern for patients. In this respect, we used the findings from a recent focus group study of people who discussed their experiences of cataract surgery.^2^ Preoperative target refraction discussions are key to good postoperative satisfaction by ensuring that the patient's visual needs are met. However, target refractive discussions have been shown to be inaccessible for patients or in some cases were not discussed at all.^2^ This focus group study also found a lack of knowledge among patients of how their final prescription would impact the type of spectacles worn postsurgery.^2^ Barriers to practitioners discussing target refraction with patients included practitioner inexperience and consultation time limitations. Optometrists with little experience were shown to be less likely to discuss target refraction when referring patients for surgery due to a lack of confidence and knowledge,^3^ suggesting that inexperienced optometrists would benefit from guidance in these clinical discussions.

Between first‐ and second‐eye surgery, patients have reported adopting a variety of coping techniques with strategies including taking a lens out of their existing spectacles, making a patch to cover their unoperated eye, wearing existing spectacles (even though vision is likely to be blurred in the operated eye) or not wearing any spectacles.^2^ Patient discussions suggested that these were trial‐and‐error approaches adopted by the patient and no best practice guidelines exist as to how practitioners should advise these patients.^2^

Many patients are motivated to undergo cataract surgery so that they can continue to drive; however, the focus group study found patients reported a huge range of information about when they could begin to drive again.^2^ Some patients recalled receiving no information, while others reported advice ranging from a few days to several weeks.^2^

The aim of this study was to develop refractive management recommendations following National Health Service (NHS) monofocal cataract surgery that have a broad agreement between optometrists and ophthalmologists. The three main topics were those highlighted by patients^2^ and were preoperative management including target refraction discussion, postoperative refractive management and particularly the interim period between first‐ and second‐eye surgery, as well as driving advice postoperatively. To develop guidelines, we adopted a Delphi consensus method. This is a well‐known method used to address issues in health for developing consensual guidance on best practice, as well as studying the feasibility of implementing recommendations.^6^, ^7^ This technique is useful when investigating a topic when there is uncertainty or limited knowledge. An advantage of the technique is that because the panellists are anonymous, this avoids any of the groups dominating the consensus process.^8^

## METHODS

**FIGURE 1:**
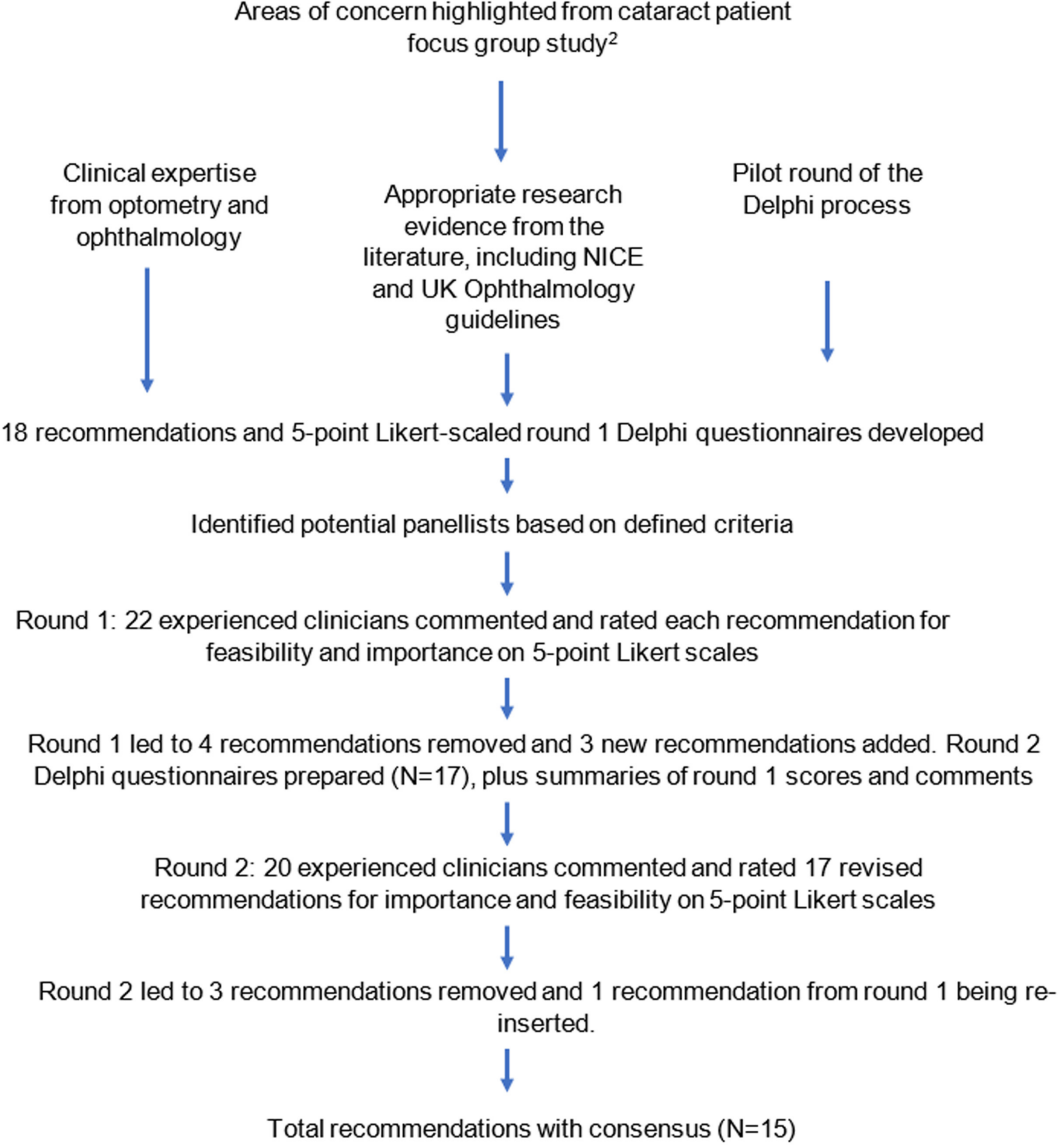
Flow chart of the survey questionnaire design and Delphi method. NICE, The National Institute for Health and Care Excellence.

The Delphi method consisted of two rounds of anonymous questionnaires that were completed between August 2021 and January 2022. The initial list of 18 recommendations (Figure 1) was developed by two optometrists (EC and DBE, 4 and 31 years of experience, respectively) and one highly experienced cataract surgeon (PU, president‐elect of the United Kingdom and Ireland Society of Cataract and Refractive Surgeons) and modified with the input from LK (highly experienced Australian optometrist, public health epidemiologist and researcher) and KCH (Hong Kong optometrist and researcher in cataract service provision). This initial list targeted areas of concern raised by patient focus groups^2^ and was evidence‐based^9^ and integrated clinical expertise from optometry and ophthalmology with the best available clinical evidence from the research literature^1^, ^2^, ^3^, ^4^, ^5^, ^7^, ^10^, ^11^, ^12^, ^13^, ^14^, ^15^, ^16^, ^17^, ^18^, ^19^, ^20^, ^21^, ^22^ including relevant sections from currently published guidelines from the UK National Institute for Health and Care Excellence (NICE)^18^ and the Royal College of Ophthalmologists^1^ (although these did not cover all of the areas of concern highlighted by patients).^2^

Recommendations were split into four categories: organisation (one item); target refraction (six items); refractive management of patients postoperatively (eight items) and driving advice following surgery (three items). The panel consisted of 11 optometrists and 11 ophthalmologists to allow both professions to have equal representation and account for any participation drop‐out. Inclusion criteria were at least 10 years of clinical experience including involvement in postcataract surgery refractive management during the past 12 months. To ensure the generalisability and expertise of the panellists, we recruited from a variety of practice settings. Optometrists were selected from multiple, independent and hospital practice, with some having University teaching experience and/or had served on Local Optical Committees and helped develop shared care schemes including direct cataract referral schemes. Ophthalmologists were all experienced surgeons conducting cataract surgery via the NHS or privately, or a mixture of the two. The study received ethics approval granted by the Chair of the Biomedical, Natural, Physical and Health Sciences Research Ethics Panel at the University of Bradford on 08 April, 2020 (EC26122).

The questionnaire was shared online using Google forms (google.com). A pilot study with five optometrists and one ophthalmologist was used to test the format and to check understanding and led to some minor rewording of the survey. The pilot included both a 5‐point and 9‐point scale with participants asked to state their scale preference. Nine‐point scales are the most frequently used in Delphi studies^7^, ^23^ followed by 5‐point scales.^7^ In an interdisciplinary context, it is not a priori clear which scale to choose for the purpose of the study.^24^ When asked during the pilot study, all six participants preferred the 5‐point scale as it was deemed more user‐friendly and was therefore adopted in the main study. A 5‐point scale had also been used successfully by this group in an earlier Delphi study.^16^ The 22 panellists were asked to rate each recommendation for feasibility and importance on the 5‐point scale (strongly disagree, disagree, neutral, agree and strongly agree) and to add comments to provide a rationale for their score, as well as to suggest any amendments to the recommendation. Panellists were also asked if they already carried out each recommendation in practice. The level of evidence supporting each recommendation was given in the brackets following each recommendation level (Level 1 = systematic reviews, Level 2 = randomised controlled trial, Level 3 = observational study, Level 4 = case studies or expert opinion). Two team members (EC and DBE) collated the feedback from each round, and all of the team members (EC, DBE, PU, KCH and LK) revised the recommendations based upon the feedback from panellists. In the second round, panellists received the amended list of recommendations and anonymised panellist's comments from round 1 along with their own scores and comments from round 1. Some recommendations which received consensus (definition of consensus given below) in round 1 underwent minor rewording to determine whether these recommendations could be improved further. Those that did not receive consensus were completely revised or removed. Amendments were highlighted to allow for easy comparison for the panellists.

The results from round 2 were then analysed, and it was determined whether a third Delphi round was required or whether sufficient consensus of the recommendations was obtained after two rounds.

### Definition of consensus

Consensus was defined based on a predetermined agreement percentage.^25^, ^26^, ^27^ The following criteria were employed^28^:

1. Consensus for a recommendation was reached if: (a) ≥80% of the panel (18/22 members) ‘moderately agreed’ and ‘strongly agreed’ for both importance and feasibility. Or (b) if consensus was reached for only one of the criteria (i.e., importance or feasibility), the other criterion could still reach consensus if ≤10% of the panel ‘strongly disagreed’ and ‘moderately disagreed’. For example, if consensus was reached for the importance of a recommendation (≥80%) but only 16/22 (73%) agreed with its feasibility, this falls below the 80% threshold and we would consider how many ‘strongly disagreed’ and ‘moderately disagreed’ with its feasibility. If 4/22 (18%) reported being neutral and 2/22 (9%) disagreed, then consensus was reached. However, if 16/22 (73%) agreed with a recommendation's feasibility, 2/22 (9%) reported being neutral and 4/22 (18%) disagreed, then consensus was not reached.
2. Recommendations without consensus in round 1 were either removed or reworded and re‐presented in round 2.

## RESULTS

A total of 22 panellists, 11 optometrists and 11 ophthalmologists, took part in the study. The optometric panel were 45% male and 55% female, and mean (SD) time qualified was 25 (10) years. Three of the optometrists had qualified as dispensing opticians prior to becoming optometrists. Six (55%) worked in independent practices, three (27%) in large multiple practices and two (18%) in the hospital eye service. In addition, two worked part‐time in university teaching and two had worked for local optical committees in developing shared care schemes including cataract referral. The ophthalmology panel was 82% male and 18% female, and mean (SD) time qualified was 17 (8) years. All ophthalmologists worked in both the NHS and private practice.

### Delphi method round 1

Round 1 consisted of 18 recommendations (Appendix S1), of which 14 (78%) reached consensus in the first round. None were rated as unimportant (i.e., ‘strongly disagree’ and ‘moderately disagree’ on importance) and/or unfeasible (i.e., ‘strongly disagree’ and ‘moderately disagree’ on feasibility). Four recommendations did not reach consensus in the first round. These were recommendation 2.5. ‘Patients due to receive large reductions in refractive error should be counselled about magnification changes’,^10^, ^11^ recommendation 3.1. ‘Patients with significant ametropia should be offered simultaneous bilateral cataract surgery to avoid anisometropia after 1st eye surgery and not obtaining a refractive correction until after 2nd eye surgery,^18^ recommendation 3.3. What time interval between 1st eye – 2nd eye surgery would you consider it NOT appropriate to recommend a new spectacle lens for the operated eye (responses varied from 2 weeks to 4 months)’ and recommendation 3.8. ‘Patients with 0.25 DC or 0.50 DC oblique astigmatism post operatively should be counselled that not including this correction would have minimal effect on vision but significantly aid adaptation to new glasses’.^10^, ^13^, ^14^ The recommendations which did not reach consensus in the first round also had low uptake in practice (with the exception of recommendation 3.3).

### Removed recommendations in round 1

Four of the recommendations in round 1 were removed (Appendix S1: 3.2, 3.5, 3.6, 3.8). Panellists were asked at what level of anisometropia they would consider recommending immediate sequential bilateral cataract surgery (ISBCS). Forty‐five per cent of optometrists would leave this to the ophthalmologist, with the rest of the advice ranging from 2.00 D to 4.00 D (2.00 D; 27%, 3.00 D; 18% and 4.00 D; 9%). Ophthalmologists' advice also ranged from 2.00 D to 4.00 D (2.00 D; 38%, 3.00 D; 50% and 4.00 D; 12%). Some stated this was not usually a deciding factor or indication for ISBCS and it may not be feasible on the NHS due to waiting list rules. It was also highlighted that high refractive error is a potential contraindication of ISBCS. These findings were incorporated into a revised round 2 recommendation.

The recommendations 3.5 and 3.6 (Appendix S1) recommended different refractive options in the interim period between first‐eye and second‐eye surgery; these included a new lens for the operated eye and using ready‐made reading glasses. Although both of these recommendations reached consensus in round 1, panellists felt multiple recommendations in the interim period were not needed as each case should be assessed individually. In round 2, interim refractive advice was condensed into one recommendation.

Recommendation 3.8: ‘Patients with 0.25 DC or 0.50 DC oblique astigmatism post operatively should be counselled that not including this correction would have minimal effect on vision but significantly aid adaptation to new glasses’^10^, ^13^, ^14^ was removed. While the literature supports this recommendation, the uptake in practice was found to be very low (18% optometrists and 10% ophthalmologists) and its importance and feasibility were also found to be low at 33% and 43%, respectively. Ophthalmologists felt this was for optometrists to discuss. Some optometrists stated that they preferred to consider this on a case‐by‐case basis and prescribe the full prescription with appropriate adaptation advice. As postoperative adaptation advice should be routine practice, it was felt this recommendation was not needed and was therefore removed.

### Added recommendations in round 1

Three new recommendations were added for round 2 (Appendix S2: 2.5; 3.2; 4.4) based on panellists' comments in round 1:

1. Round 2, 2.5: ‘Non‐NHS options including multifocal, toric and extended depth‐of‐focus intraocular lenses (IOLs) should be discussed with appropriate patients.’ Ophthalmologists providing feedback for the recommendations regarding the myopic patients being offered a myopic target refraction and patients using a monovision approach offered a monovision target refraction commented how, ‘Such patients should also be informed about extended depth of focus and multifocal lens implants’ (Ophthalmologist—panellist 6) and ‘Monovision, toric, multifocal lenses and extended depth of vision lenses are all options that are available on the market and need to be discussed’ (Ophthalmologist—panellist 2). Therefore, this was added as a recommendation in round 2.
2. Round 2, 3.2: ‘A range of refractive options should be discussed with patients between eye monofocal IOL surgeries. These include: (i) a new spectacle lens for the operated eye. (ii) Removing the lens of the operated eye from their glasses. (iii) Abandoning glasses and using ready‐made readers’.^1^, ^7^ During round 1, the list of refractive options in the interim period between the first‐eye and second‐eye surgery were listed as separate recommendations. Both optometrists and ophthalmologists felt the management of this period should be assessed individually and involve a discussion with the patient rather than recommending specific advice, ‘Again individually assessed. How are they coping between surgeries? Would they personally benefit from a temporary correction? Only a full dispensing case history with patient inclusion will allow a successful decision’ (Optometrist—panellist 4). Most felt, ‘This is a judgement call for the optometrist and does not need guidance’ (Optometrist—panellist 7). Rather than recommending each refractive option separately, we condensed them into one recommendation stating a range of refractive options should be discussed in the interim period.
3. Round 2, 4.4: ‘Patients with further concerns about driving should be advised to see their optometrist.’ Feedback from the driving advice recommendations raised concerns that patient's judgement on the level of vision needed for driving is unreliable. Optometrists commented that they were happy to check visual acuities postoperatively to reassure the patient. ‘Patients should be advised that they can drive following surgery if they feel confident and can see registration plate at the appropriate distance but should be able to ask their post‐op optometrist ahead of schedule if concerned’ (Optometrist—panellist 6). It was added as a recommendation that patients with further concerns about driving should see their optometrist.

### Delphi method round 2

After the removal and addition of new recommendations, the remaining recommendations underwent minor rewording amendments based upon the feedback and comments received from round 1. Twenty panellists took part in round 2 as two ophthalmologists did not respond during the second round. During the second round, recommendations were rerated on importance and feasibility and 14 of 17 (82%) recommendations reached consensus.

Three recommendations did not reach consensus in the second round. Recommendation 3.1 ‘Patients with significant ametropia should be offered simultaneous bilateral cataract surgery to avoid anisometropia after 1st eye surgery and not obtaining a refractive correction until after 2nd eye surgery’ was updated following round 1 advice. Panellists felt bilateral surgery would be useful for patients to avoid anisometropia between the first‐ and second‐eye surgery. However, there were concerns that significant ametropia involving significantly short or long eyes would be a contraindication for ISBCS. When panellists were asked at what level of anisometropia they would consider ISBCS, the majority said 2.00 D. Therefore, in round 2, a requirement of ametropia between 2.00 D and 6.00 D was added to the recommendation. Although both importance (64%–89%) and feasibility (38%–78%) improved the recommendation, it still did not achieve consensus due to slightly low feasibility (2% below the cut‐off). While panellists considered that this was important, many ophthalmologists felt it was not currently feasible in the NHS system. As this recommendation did not achieve consensus after two rounds, it was removed from the final list of recommendations.

Recommendation 3.3 (round 1) investigated at what time interval between the first eye and second eye surgery would you consider it NOT appropriate to recommend a new spectacle lens for the operated eye. During round 1, optometrists and ophthalmologists could select between <2 weeks, <1 month, <2 months, <4 months, <8 months and 1 year+. Fourteen per cent opted <2 weeks, 48% opted <1 month, 28% opted 2 months and 10% opted <4 months. As 1 month was the most popular response in round 2, the recommendation was updated to ‘3.3. A time interval between 1st eye – 2nd eye surgery of <1 month should typically be considered too short to recommend a new spectacle lens for the operated eye’. Ophthalmologists had agreement on both importance (89%) and feasibility (86%). However, optometrists had less agreement, with 70% agreeing it was important and 56% agreeing it was feasible. Most optometrists commented that they felt this was patient dependent and depends upon their visual needs. Some patients are happy to update as a temporary solution, whereas others stated that in their experience patients prefer to wait until they have had the second‐eye surgery before updating their spectacles. As this recommendation did not achieve consensus after two rounds, it was removed from the final list of recommendations.

Recommendation 4.1 was updated (4.2 in round 2) following round 1 advice. Panellists suggested that this recommendation regarding driving advice could be expanded further to include, ‘do they have double vision and can they judge depth correctly’ (Ophthalmologist—panellist 10). Others mentioned suggesting a specific time period to allow for adaptation, ‘It is advisable to suggest allowing 5‐7 days after surgery to allow for some adaptation’ (Ophthalmologist—panellist 2). A time period of 1 week and information regarding double vision was included in the next round. However, in round 2, its importance dropped from 82% to 75% and feasibility dropped from 79% to 77%. Concerns remained regarding patient judgement on the level of vision required to reach the UK driving standards. Ophthalmologists expressed concerns, ‘Who will measure 20.5 m to read a number plate? This sort of advice should be avoided to avoid litigation’ (Ophthalmologist—panellist 6) and that ‘The period of 1 week is overly conservative and not based in evidence’ (Ophthalmologist—panellist 10). Optometrists also expressed concerns over the recommendation and felt patients self‐measuring their vision was unreliable. ‘Confidence is not a measure applicable to the Highway Code, and we have all had the patient who was still driving at 6/60 R and L who claimed to have no problem with their distance vision’ (Optometrist—panellist 2). As the recommendation reached consensus in round 1 but not in round 2, the recommendation was reverted back to the original wording. After two rounds, 15 recommendations were finalised (Table 1).

**TABLE 1:** Showing the final 15 recommendations which reached consensus after two rounds of the Delphi method.

	Recommendation
Organisation	
1.1	Joint refractive management can provide high‐quality and convenient patient care if agreed protocols and appropriate training and remuneration are provided within a commissioned shared care system
Target refractive errors	
2.1	The patient must be fully informed prior to any decisions regarding their postoperative target refractive error, including issues of IOL type, and how this will affect the cost and convenience of postoperative spectacle wear, if needed (level 3–4 evidence)^2^, ^15^
2.2	To fully inform the patient, an ideal process is an initial discussion by the referring optometrist to introduce the idea of refractive outcomes and outline options with further discussion with the ophthalmologist to clarify understanding and make a decision (level 3–4)^2^, ^15^
2.3	Myopic patients used to reading without glasses should particularly be made aware of the option of a myopic target refraction in NHS (monofocal IOL) referrals (level 3–4 evidence)^2^, ^15^, ^16^, ^34^, ^35^
2.4	Patients with a history of using a monovision approach should be made aware that this approach could be provided postsurgery, particularly in NHS (monofocal IOL) referrals (level 4 evidence)^15^, ^36^
2.5	Non‐NHS options including multifocal, toric and extended depth‐of‐focus IOLs should be discussed with appropriate patients
2.6	Patients due to receive reductions in refractive error >2.00 D should be counselled about the potential for anisometropic symptoms between first‐ and second‐eye surgery (level 3–4 evidence)^10^, ^11^
2.7	Patients should be provided with both verbal and written advice about their target refraction prior to surgery
Refractive management of patients	
3.1	A range of refractive options should be discussed with patients between eye monofocal IOL surgeries. These include: (i) a new spectacle lens for the operated eye. (ii) Removing the lens of the operated eye from their glasses. (iii) Abandoning glasses and using ready readers (level 3 & 4 evidence)^2^, ^10^
3.2	The benefits and cost of prescribing a single vision balance lens (or a contact lens) to the unoperated second eye should be discussed with patients struggling with anisometropia post‐first eye surgery (level 4 evidence)^10^, ^13^
3.3	Patients who have had uncomplicated surgery and urgently require new spectacles (e.g., for driving) should be offered the option of updated spectacles before the current guidelines of 4–6 weeks with potential cost implications discussed (level 1, 3 and 4)^37^
Driving advice following surgery	
4.1	Patients should be provided with both verbal and written driving advice following surgery, ideally as part of the information about the surgery
4.2	Patients should be advised that they can drive following surgery if they feel confident and can see a registration plate at the appropriate distance (level 4 evidence) (The Royal College of Surgeons of England, 2022)^38^
4.3	Patients with surgery‐induced anisometropia and/or loss of stereopsis should be advised that this may cause problems with driving and may require a longer period of adaptation (level 3–4 evidence)^17^, ^32^
4.4	Patients with further concerns about driving should be advised to see their optometrist

Abbreviations: IOL, intraocular lens; NHS, National Health Service.

### Follow‐up questions

Both optometrists and ophthalmologists were asked what percentage of their cataract patients were referred directly to the Hospital Eye Service. Of the 16 (out of 22 panellists) who responded, the median response was 75% (10%–90% interquartile range). All panellists were asked if they believed optometrists should play a role in the provision of information regarding target refraction and postoperative refractive management of patients with cataract. All panellists replied positively with the exception of one ophthalmologist (i.e., 21/22, 95%).

## DISCUSSION

A Delphi method was used to achieve consensus on recommendations for the pre and postoperative refractive management of cataract surgery patients. Fifteen recommendations reached consensus after two rounds of the Delphi method. Agreed recommendations between both optometrists and ophthalmologists can improve the outcome of cataract surgery for patients by ensuring that they are provided with the knowledge to make informed decisions regarding their surgery and refractive management.

One hundred per cent of optometrists and 91% of ophthalmologists believed optometrists should play a role in the provision of information regarding target refraction and postoperative refractive management of patients with cataract. All panellists agreed that all patients must be informed prior to any decision regarding their postoperative target refraction. Ophthalmologists stated that the depth of discussion is limited by clinic time and would be improved if the process of discussing options began before the patient arrived in the hospital. Optometrists agreed that starting the process of discussing target refraction at the point of referral would make the presurgical assessment more efficient, and of particular benefit to myopic and monovision patients. However, time constraints and the surgeon making the final decision on target refraction may make some optometrists reluctant to undertake target refraction discussions. This could be overcome by providing appropriate training for optometrists, ideally with the support of local ophthalmologists. While the suggestion of providing patients with both verbal and written target refraction advice achieved high importance (91%) and feasibility (82%) in round 1, suggesting a patient leaflet could be developed between optometry and ophthalmology feasibility dropped to 70%, and therefore, this was removed from the recommendations. Panellists felt it would be difficult to envisage a single leaflet due to various permutations and patient's different levels of understanding. Including patients in the development of information leaflets may be needed to ensure the material is relevant, readable and understandable to patients.^18^

Three further recommendations achieved low feasibility (round 2, 2.5; 3.1; 4.2; Appendix S2). The first was related to discussing non‐NHS options including multifocal, toric and extended depth‐of‐focus IOLs with appropriate patients. While panellists agreed this was crucial and part of the informed consent process, opinions differed as to when this should first be discussed with the patient. Most believed this should first be discussed by the optometrist before referral to the NHS, although one optometrist raised concerns that more training is required as they felt they had limited knowledge.

### Immediate sequential bilateral cataract surgery

The second recommendation to achieve low feasibility discussed offering ISBCS to patients with no contraindications and 2.00–6.00 D of ametropia in order to avoid anisometropia between the first‐ and second‐eye surgeries. There was strong agreement of 90% with panellists believing it would be hugely beneficial for patients to avoid anisometropic symptoms,^19^ negate many adaptation problems and potentially reduce the number of injurious falls in this period.^12^ Ophthalmologists highlighted that practice is slowly moving towards ISBCS, although this technique is not currently feasible within all NHS organisations due to strict preoperative protocols, the modern and accurate biometry devices needed to reduce refractive surprise and not all surgeons being comfortable with the technique. NICE recommended that ISBCS should be considered for two groups of patients: (1) those at low risk of operative and postoperative complications, (2) those who need to have general anaesthesia for the surgery but for whom general anaesthesia carries an increased risk of complications or distress.^15^ The vast majority of cataract surgery patients are at low risk of operative and postoperative complications. However, it was found that the technique is predominantly performed under general anaesthetic (60%) and in more complex patients with issues lying flat, cooperating, mature cataracts and no fundal view.^20^ Only 0.5% of UK cataract operations were performed as ISBCS between 2010 and 2018.^20^ Due to the COVID‐19 pandemic, cataract services were redesigned.^4^ The Royal College of Ophthalmologists and the UK and Ireland Society of Cataract and Refractive Surgery jointly recommended more ISBCS in suitable patients to streamline cataract services^21^ and reduce the risk of COVID‐19 transmission in hospital.^22^, ^29^ One NHS hospital which published their ISBCS figures found an increase from 0.36% in the 5 years before COVID‐19 to 3.05% in the 6 months following the reopening of cataract services after closure due to the pandemic.^30^ It is evident that current cataract services will have to evolve due to the ageing population in the UK.^31^ In the long term, continued and increased uptake of ISBCS may be advantageous and would be beneficial to the patient in achieving full quality of life gains faster. A prolonged gap between the first‐ and second‐eye surgery has been shown to increase the risk of falls^12^ and the number of ‘head on’ and ‘hit pedestrian’ driving accidents.^32^ Removing the interim period between the first‐ and second‐eye surgeries will eliminate anisometropic symptoms and subsequent aniseikonia for the patient, potentially reducing the risk of falls, car crashes and their associated healthcare costs. As this recommendation was extremely close to consensus (78% feasible and 11% nonfeasible with consensus figures of 80% or 10%), it is possible that a third round of the Delphi study could have reached consensus with appropriate rewording, and a recommendation regarding ISBCS should be added to any guidelines in the near future should its use increase significantly.

### Driving advice

The third recommendation to achieve low feasibility was regarding how soon patients should be advised they can drive following surgery. Currently, no recommendations for driving postsurgery exist. It has been shown that the postoperative driving advice that patients receive is limited or nonexistent and varies from a few days to a few weeks.^2^ During round 1, it was suggested by ophthalmologists to include a time period of 5–7 days to allow for adaptation and to expand the recommendation to include a comment about double vision. This reduced importance and feasibility to 75% and 74%. Round 2 feedback concluded a time period of 1 week is overly conservative, not based on evidence, and the recommendation should be less restrictive. There were also concerns of patients not accurately measuring the distance to read a number plate in order to satisfy the UK Driver and Vehicle Licensing Agency (DVLA) driving standards. It is important that all patients are provided with postoperative driving advice and are made aware that it is their responsibility to ensure they meet the driving standards. Patients being provided with both verbal and written driving advice following surgery achieved 90% importance and feasibility. It may be helpful to include the driving standards in a postoperative leaflet provided to the patient, as it has been shown that the vast majority of patients do not know the DVLA driving standards.^33^ Any patients with concerns as to whether they meet the driving standards should be advised to see their optometrist for a vision check.

Two recommendations did not reach consensus after two rounds of the Delphi method (Appendix S2: 3.1; 3.3). Recommendation 3.3 discussed the time interval required before advising a new spectacles lens between the first‐ and second‐eye surgery. Panellists felt this was primarily a cost issue. While ophthalmologists rated the recommendation of high importance (89%) and feasibility (86%), optometrists felt that a recommendation was too prescriptive to execute in the real world (importance 70% and feasibility 60%). It was felt that the refractive advice required is very patient dependent, and practitioners may need to deviate from such a recommendation to meet a patient's needs and available budget.

### Strengths and limitations

A strength of the study was that it used a patient‐centred approach, and the recommendations were based on areas of concern for patients. In addition, the multidisciplinary panel of optometrists and ophthalmologists had at least 10 years of experience working in a range of settings. Although not representative of UK optometry and ophthalmology practitioners, they were very experienced and well‐informed clinicians. One limitation of the study may be that panellists who volunteered to take part may have a particular interest in the pre and postoperative refractive management of cataract surgery patients and perhaps were more positive about collaboration between optometrists and ophthalmologists. A further limitation could be that a third round could have been used to achieve consensus on the recommendations that did not achieve consensus and further explore those that achieved low feasibility. However, due to time limitations, difficulty of obtaining some responses and the reduced number of respondents for round 2, this was deemed not viable.

## CONCLUSION

Fifteen recommendations reached consensus after two rounds of the Delphi method using a panel of experienced optometrists and ophthalmologists. They support optometrists playing a role in the provision of information regarding target refraction and postoperative refractive management of patients with cataract. Implementing these recommendations in practice could both remediate areas of concern for patients,^2^ help optometrists with their clinical decisions and discussions and may be of particular benefit to less experienced optometrists.^3^ Joint refractive management by optometrists and ophthalmologists could provide high‐quality and convenient patient care if agreed protocols and appropriate training and remuneration were provided.

## AUTHOR CONTRIBUTIONS

**Emily Charlesworth:** Conceptualization (equal); data curation (equal); formal analysis (equal); investigation (equal); methodology (equal); project administration (equal); writing – original draft (lead); writing – review and editing (equal). **Paul Ursell:** Conceptualization (equal); data curation (equal); formal analysis (equal); investigation (equal); methodology (equal); project administration (equal); writing – original draft (supporting); writing – review and editing (equal). **Kam Chun Ho:** Conceptualization (equal); data curation (equal); formal analysis (equal); investigation (equal); methodology (equal); writing – original draft (supporting); writing – review and editing (equal). **Lisa Keay:** Conceptualization (equal); data curation (equal); formal analysis (equal); investigation (equal); methodology (equal); writing – original draft (supporting); writing – review and editing (equal). **David B. Elliott:** Conceptualization (equal); data curation (equal); formal analysis (equal); investigation (equal); methodology (equal); project administration (equal); supervision (lead); validation (equal); writing – original draft (equal); writing – review and editing (equal).

## CONFLICTS OF INTEREST

The authors declare that there are no conflicts of interest.

## Supporting information


Appendix S1.

Appendix S2.
