# Supplementary material for: Developing refractive management recommendations for patients undergoing cataract surgery: A Delphi study
Source: Ophthalmic Physiol Opt. 2022 Nov 16;43(1):150–9. doi: 10.1111/opo.13069 (PMC10100233; doi:10.1111/opo.13069)
Supplement: Supplementary file 1 — Appendix S1. [file OPO-43-150-s002.docx]

| Appendix 1: showing the initial 18 recommendations presented in round 1 of the Delphi method. Those recommendations in bold did not reach consensus in first round. Red percentages indicated those <80%. If only one of the criteria (i.e., importance or feasibility) was rated >80% the other criterion must have ≤ 10% of the panel rated on the opposite side of agreement. | **Optometrists - % already do in practice** | **Ophthalmologists - % already do in practice** | **Importance** | **Feasibility** | **Recommendation** |
| --- | --- | --- | --- | --- | --- |
| **Organisation** |  |  |  |  |  |
| **1.1** | 55% | 45% | 100%  N=22/22 | 91%  N=22/22 | Joint management can provide the best patient care if agreed protocols and appropriate training and remuneration are provided within a shared care system. |
| **Target refractive errors** |  |  |  |  |  |
| **2.1** | 64% | 100% | 100%  N=22/22 | 82%  N=18/22 | The patient must be fully informed prior to any decisions regarding their post-operative target refractive error, including issues of cost and convenience (level 3-4 evidence) ^2, 21^. |
| **2.2** | 60% | 55% | 86%  N=19/22 | 82%  N=19/22 | To fully inform the patient discussions should typically involve an initial preliminary discussion by the optometrist with further discussion with the ophthalmologist (level 3-4) ^2, 21^. |
| **2.3** | 60% | 100% | 95%  N=21/22 | 95%  N=21/22 | Myopic patients used to reading without glasses should be made aware of the option of a myopic target refraction (level 3-4 evidence) ^2, 21-24^. |
| **2.4** | 70% | 100% | 100%  N=22/22 | 86%  N=19/22 | Patients using a monovision approach should be made aware that this approach could be provided post-surgery (level 4 evidence) ^21, 25^. |
| **2.5** | 40% | 64% | **73%**  **N=16/22** | **73%**  **N=16/22** | **Patients due to receive large reductions in refractive error should be counselled about magnification changes (level 3-4 evidence) ^16, 17^.** |
| **2.6** | 18% | 91% | 91%  N=20/22 | 82%  N=18/22 | Patients should be provided with both verbal and written advice about their target refraction prior to surgery. |
| **Refractive management of patients** |  |  |  |  |  |
| **3.1** | 10% | 45% | **64%**  **N=14/22** | **36%**  **N=8/22** | **Patients with significant ametropia should be offered simultaneous bilateral cataract surgery to avoid anisometropia after 1^st^ eye surgery and not obtaining a refractive correction until after 2^nd^ eye surgery (level 3 Evidence) ^18^.** |
| **3.2** | N/A | N/A | N/A | N/A | *What level of anisometropia would you consider suggests the recommendation to the patient of bilateral simultaneous surgery.* |
| **3.3** | N/A | N/A | N/A | N/A | ***What time interval between 1^st^ eye – 2^nd^ eye surgery would you consider it NOT appropriate to recommend a new spectacle lens for the operated eye.*** |
| **3.4** | 82% | 60% | 90%  N=19/21 | 90%  N=19/21 | Patients with anisometropia post 1^st^ eye surgery should be counselled about the benefits and cost of prescribing a single vision balance lens (or a contact lens) to the unoperated 2^nd^ eye (level 4 evidence) ^16, 19^. |
| **3.5** | 82% | 80% | 80%  N=16/20 | 80%  N=16/20 | Patients who intend to wear distance glasses after 2nd eye surgery should be counselled about the benefits and cost of a new spectacle lens for the operated eye between surgeries (level 3 evidence) ^16^. |
| **3.6** | 64% | 100% | 85%  N=17/20 | 90%  N=18/20 | Patients who do not intend to wear distance glasses after 2nd eye surgery should be counselled about the benefits and cost of not using current spectacles during the interim period and using ready readers for near vision (level 4) ^2^. |
| **3.7** | 64% | 50% | 81%  N=17/21 | 67%  N=14/21  Not feasible 9.5% | Patients who have had uncomplicated surgery and urgently require new spectacles (e.g., for driving) should be offered the option of updated spectacles before the current guidelines of 4-6 weeks (level 1, 3 and 4) ^26^. |
| **3.8** | 18% | 10% | **33%**  **N=7/21** | **48%**  **N=10/21** | **Patients with 0.25DC or 0.50DC oblique astigmatism post operatively should be counselled that not including this correction would have minimal effect on vision but significantly aid adaptation to new glasses (level 3 evidence) ^16, 19, 20^.** |
| **Driving advice following surgery** |  |  |  |  |  |
| **4.1** | **73%** | **91%** | 82%  N=18/22 | 77%  N=17/22  Not feasible 9% | Patients should be advised that they can drive following surgery if they feel confident and can see registration plate at the appropriate distance (level 4 evidence). |
| **4.2** | 70% | 82% | 91%  N=20/22 | 91%  N=20/22 | Patients with anisometropia and/or loss of stereopsis should be advised that this may cause problems with driving such as judging speed and distances (level 3-4 evidence) ^28, 29^. |
| **4.3** | 18% | 73% | 82%  N=18/22 | 82%  N=18/22 | Patients should be provided with both verbal and written driving advice following surgery. |
