# Supplementary material for: Developing refractive management recommendations for patients undergoing cataract surgery: A Delphi study
Source: Ophthalmic Physiol Opt. 2022 Nov 16;43(1):150–9. doi: 10.1111/opo.13069 (PMC10100233; doi:10.1111/opo.13069)
Supplement: Supplementary file 2 — Appendix S2. [file OPO-43-150-s001.docx]

| Appendix 2: showing the 17 recommendations presented in round 2 of the survey. Amendments to the recommendation are shown in red. Those in bold did not reach consensus. Red percentages indicated those <80%. If only one of the criteria (i.e., importance or feasibility) was rated >80% the other criterion must have ≤ 10% of the panel rated on the opposite side of agreement. | **Round 1**  **Importance** | **Round 1**  **Feasibility** | **Round 2**  **Importance** | **Round 2**  **Feasibility** | **Recommendation** |
| --- | --- | --- | --- | --- | --- |
| **Organisation** |  |  |  |  |  |
| **1.1** | 100%  N=22/22 | 91%  N=22/22 | 100%  N=20/20 | 85%  N=17/20 | Joint refractive management can provide high quality and convenient patient care if agreed protocols and appropriate training and remuneration are provided within a commissioned shared care system. |
| **Target refractive errors** |  |  |  |  |  |
| **2.1** | 100%  N=22/22 | 82%  N=18/22 | 100%  N=20/20 | 80%  N=16/20 | The patient must be fully informed prior to any decisions regarding their post-operative target refractive error, including issues of IOL type, and how this will affect the cost and convenience of post-operative spectacle wear, if needed (level 3-4 evidence) ^2, 21^. |
| **2.2** | 86%  N=19/22 | 82%  N=19/22 | 100%  N=20/20 | 85%  N=17/20 | To fully inform the patient, an ideal process is an initial discussion by the referring optometrist to introduce the idea of refractive outcomes and outline options with further discussion with the ophthalmologist to clarify understanding and make a decision (level 3-4) ^2, 21^. |
| **2.3** | 95%  N=21/22 | 95%  N=21/22 | 100%  N=20/20 | 90%  N=18/20 | Myopic patients used to reading without glasses should particularly be made aware of the option of a myopic target refraction in NHS (monofocal IOL) referrals (level 3-4 evidence) ^2, 21-24^. |
| **2.4** | 100%  N=22/22 | 86%  N=19/22 | 95%  N=19/20 | 90%  N=18/20 | Patients with a history of using a monovision approach should be made aware that this approach could be provided post-surgery, particularly in NHS (monofocal IOL) referrals (level 4 evidence) ^21, 25^. |
| **2.5** | N/A | N/A | 95%  N=19/20 | *75%*  *N=15/20*  Not feasible 10% (2/20) | Non-NHS options including multifocal, toric and extended depth-of-focus IOLs should be discussed with appropriate patients. |
| **2.6** | 73%  N=16/22 | 73%  N=16/22 | 100%  N=20/20 | 100%  N=20/20 | Patients due to receive reductions in refractive error greater than 2.00D should be counselled about the potential for anisometropic symptoms between first and second eye surgery (level 3-4 evidence) ^16, 17^. |
| **2.7** | 91%  N=20/22 | 82%  N=18/22 | 85%  N=17/20 | *70%*  *N=14/20*  Not feasible 10% (2/20) | Patients should be provided with both verbal and written advice about their target refraction prior to surgery. Ideally the latter should be a patient information leaflet developed by an ophthalmology and optometry team. |
| **Refractive management of patients** |  |  |  |  |  |
| **3.1** | 64%  N=14/22 | 36%  N=8/22 | 89%  N=17/19 | 78%  N=14/18  Not feasible 11% (2/18) | If there are no contra-indications, patients with ametropia between approximately 2.00 to 6.00D could be offered immediate sequential bilateral cataract surgery (ISBCS) to avoid anisometropia after 1st eye surgery and not obtaining a refractive correction until after 2nd eye surgery (Level 3 Evidence) ^18^. |
| **3.2** | N/A | N/A | 100%  N=20/20 | 100%  N=20/20 | A range of refractive options should be discussed with patients between eye monofocal IOL surgeries. These include (i) a new spectacle lens for the operated eye. (ii) Removing the lens of the operated eye from their glasses. (iii) Abandoning glasses and using ready readers (level 3 & 4 evidence) ^16, 19^. |
| **3.3** | N/A | N/A | 79%  N=15/19 | 71%  N=12/17 | A time interval between 1st eye – 2nd eye surgery of less than 1 month should typically be considered too short to recommend a new spectacle lens for the operated eye. |
| **3.4** | 90%  N=19/21 | 90%  N=19/21 | 80%  N=16/20 | 80%  N=16/20 | The benefits and cost of prescribing a single vision balance lens (or a contact lens) to the unoperated 2nd eye should be discussed with patients struggling with anisometropia post 1st eye surgery (level 4 evidence) ^16, 19^. |
| **3.5** | 81%  N=17/21 | 67%  N=14/21  Not feasible 9.5% | 90%  N=18/20 | 95%  N=18/20 | Patients who have had uncomplicated surgery and urgently require new spectacles (e.g., for driving) should be offered the option of updated spectacles before the current guidelines of 4-6 weeks with potential cost implications discussed (level 1, 3 and 4) ^26^. |
| **Driving advice following surgery** |  |  |  |  |  |
| **4.1** | 82%  N=18/22 | 82%  N=18/22 | 90%  N=18/20 | 90%  N=18/20 | Patients should be provided with both verbal and written driving advice following surgery, ideally as part of the information about the surgery. |
| **4.2** | 82%  N=18/22 | *77%*  *N=17/22*  Not feasible 9% | 75%  N=15/20 | 74%  N=14/19 | Patients should be advised that they can drive to their level of confidence approximately 1 week following surgery if they can see a registration plate at the appropriate distance and do not have any double vision (level 4 evidence). |
| **4.3** | 91%  N=20/22 | 91%  N=20/22 | 100%  N=20/20 | 100%  N=20/20 | Patients with surgery-induced anisometropia and/or loss of stereopsis should be advised that this may cause problems with driving and may require a longer period of adaptation (level 3-4 evidence) ^28, 29^. |
| **4.4** | N/A | N/A | 100%  N=20/20 | 95%  N=18/19 | Patients with further concerns about driving should be advised to see their optometrist. |
